# Bilateral chylothorax following total thyroidectomy with neck lymph node dissection for thyroid cancer: a case report and literature review

**DOI:** 10.3389/fonc.2024.1489410

**Published:** 2025-01-08

**Authors:** Yunsheng Wang, Xudong Liu, Xingyue Wang, Youxin Tian, Qinjiang Liu, Jun Wang, Jincai Xue

**Affiliations:** Department of Head and Neck Surgery, Gansu Provincial Cancer Hospital, Lanzhou, China

**Keywords:** chylothorax, thyroid cancer, neck lymph node dissection, postoperative complications, case report

## Abstract

**Purpose:**

Investigating the diagnosis and treatment of bilateral Chylothorax after neck lymph node dissection for thyroid cancer.

**Methods:**

The clinical data of a patient with bilateral chylothorax after neck lymph node dissection for thyroid cancer were retrospectively analyzed, and the relevant literature was reviewed.

**Results:**

The patient underwent a total thyroidectomy and left neck lymph node dissection, with no evidence of lymph fluid leakage observed during the operation. The patient experienced chest tightness, shortness of breath, dyspnea, and decreased lung auscultation breath sounds on the 7th day after the surgery. The chest X-ray examination revealed the presence of bilateral pleural effusion. Under ultrasound guidance, bilateral thoracic closed drainage tube was implanted, and a small sample of the milky white fluid was tested for chylothorax, yielded positive results. The patient is diagnosed with bilateral chylothorax. After received conservative treatment, the patient’s drainage flow gradually decreased. Subsequent review of a chest X-ray showed no signs of chest hydrops, and as a result, the thoracic drainage tube was removed. The patient eventually recovered and was subsequently discharged.

**Conclusion:**

Bilateral chylothorax is a rare complication following neck lymph node dissection for thyroid cancer. It is deemed safe and effective to administer active conservative treatment upon early detection.

## Introduction

Thyroid cancer is a prevalent endocrine malignancy that has shown an increasing incidence in recent years (1, 2). When thyroid cancer is accompanied by cervical lymph node metastasis, it necessitates cervical lymph node dissection. The dissection should encompass areas II, III, IV, and V (3). Chyle leakage is a serious complication following cervical lymph dissection for thyroid cancer. The reported incidence of chyle leakage is approximately 1% to 2.5% (4). However, the occurrence of chylothorax following cervical lymph node dissection is rare, particularly bilateral chylothorax (4). Since Stuart first reported bilateral chylothorax after cervical lymph dissection in 1907, there have been mainly reported cases so far (5). We report a case of bilateral chylothorax after left neck lymph node dissection for thyroid cancer.

## Case report

**Figure 1:**
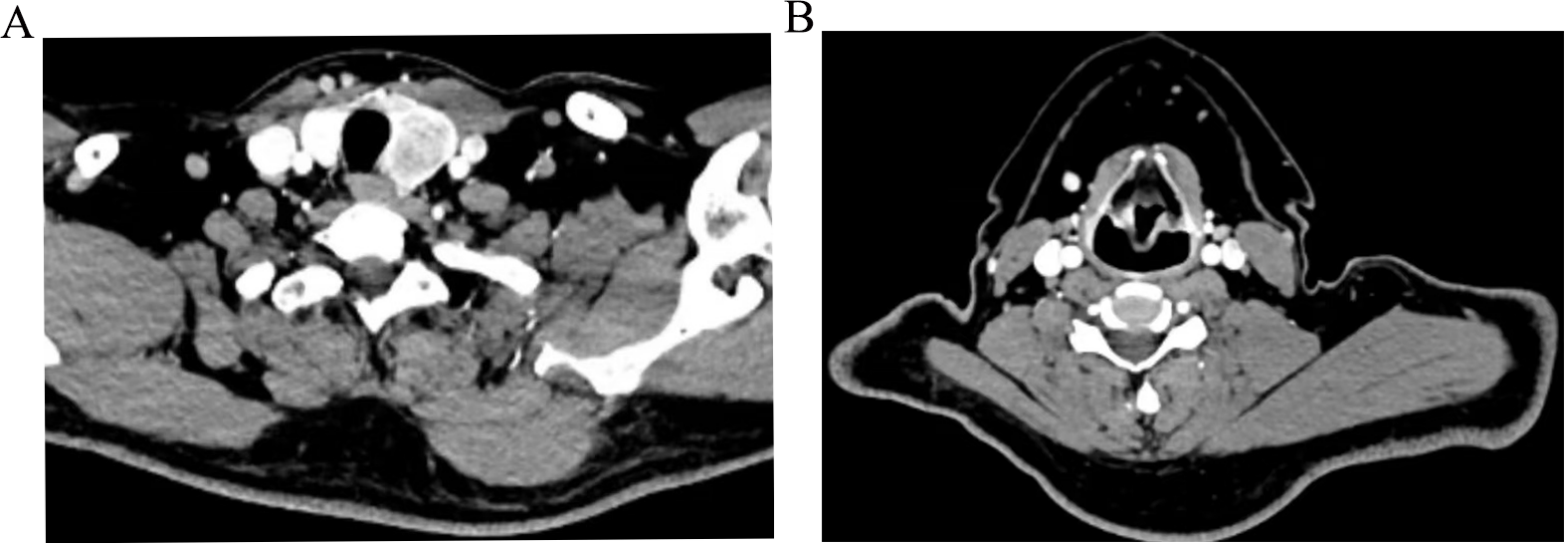
Preoperative CT images of the left thyroid **(A)**, metastatic lymph nodes in the left neck region III **(B)**.

The patient is a 40-year-old male who was admitted to the hospital due to the discovery of left thyroid nodules during a physical examination that had been present for more than two months. The patient denies any past medical history of diseases, surgeries, or allergies. Physical examination: A palpable mass of about 4.0cm×1.5cm with a hard texture and unclear boundary is present in the left thyroid lobe. Additionally, multiple enlarged lymph nodes are detected on the left side of the neck, with the larger one located in the III region measuring about 2.0cm×2.0cm and exhibiting a hard quality and unclear boundary. The remainder of the site does not exhibit any apparent mass (**Figure 1**). Ultrasonography of the thyroid gland revealed a hypoechoic mass in the left lobe, measuring approximately 4.4cm×1.2cm with an irregular shape. The Thyroid Imaging Reporting and Data System (TI-RADS) score classified the left nodule as grade V, indicating a high suspicion for malignancy. Additionally, enlargement of the left cervical lymph node was deemed to be metastatic. There was no apparent lesion found in the right lobe of the thyroid gland. Fine needle puncture pathology revealed left papillary thyroid carcinoma and a left neck mass consistent with lymph node metastasis of papillary thyroid carcinoma. Fine-needle aspiration revealed left papillary thyroid carcinoma and a left neck mass consistent with lymph node metastasis of papillary thyroid carcinoma. Preoperative chest X-rays revealed normal findings. Clinical diagnosis: Left thyroid cancer with left cervical metastasis (cT2N1bM0 stage I). The routine preoperative examination revealed no contraindications. On July 30, 2019, total thyroidectomy and left neck lymph node dissection in areas II, III, IV, V, and VI were performed under general anesthesia. The thoracic duct was not routinely exposed during the operation, and the anesthesiologist changed the ventilation mode to manual control and increased airway pressure to temporarily increase venous pressure after the operation, and no lymphatic exudation or gelatinous object formation was observed. A drainage tube was placed into the operative area to facilitate negative pressure drainage.

**Figure 2:**
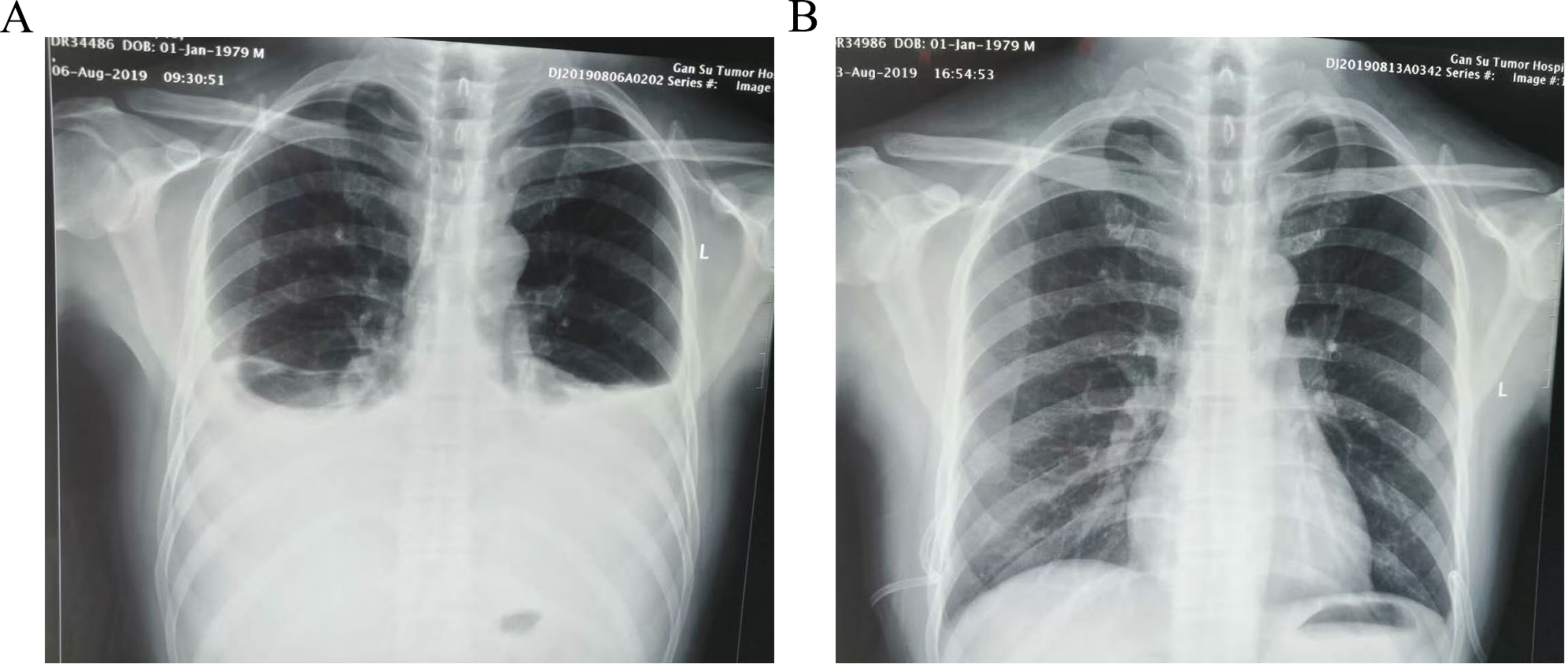
Chest X-ray images of patient before and after treatment **(A)**:a chest radiograph revealed bilateral pleural effusion on postoperative day 7. **(B)** No obvious thoracic fluid collections in the review X-ray image on postoperative day 14.

**Figure 3:**
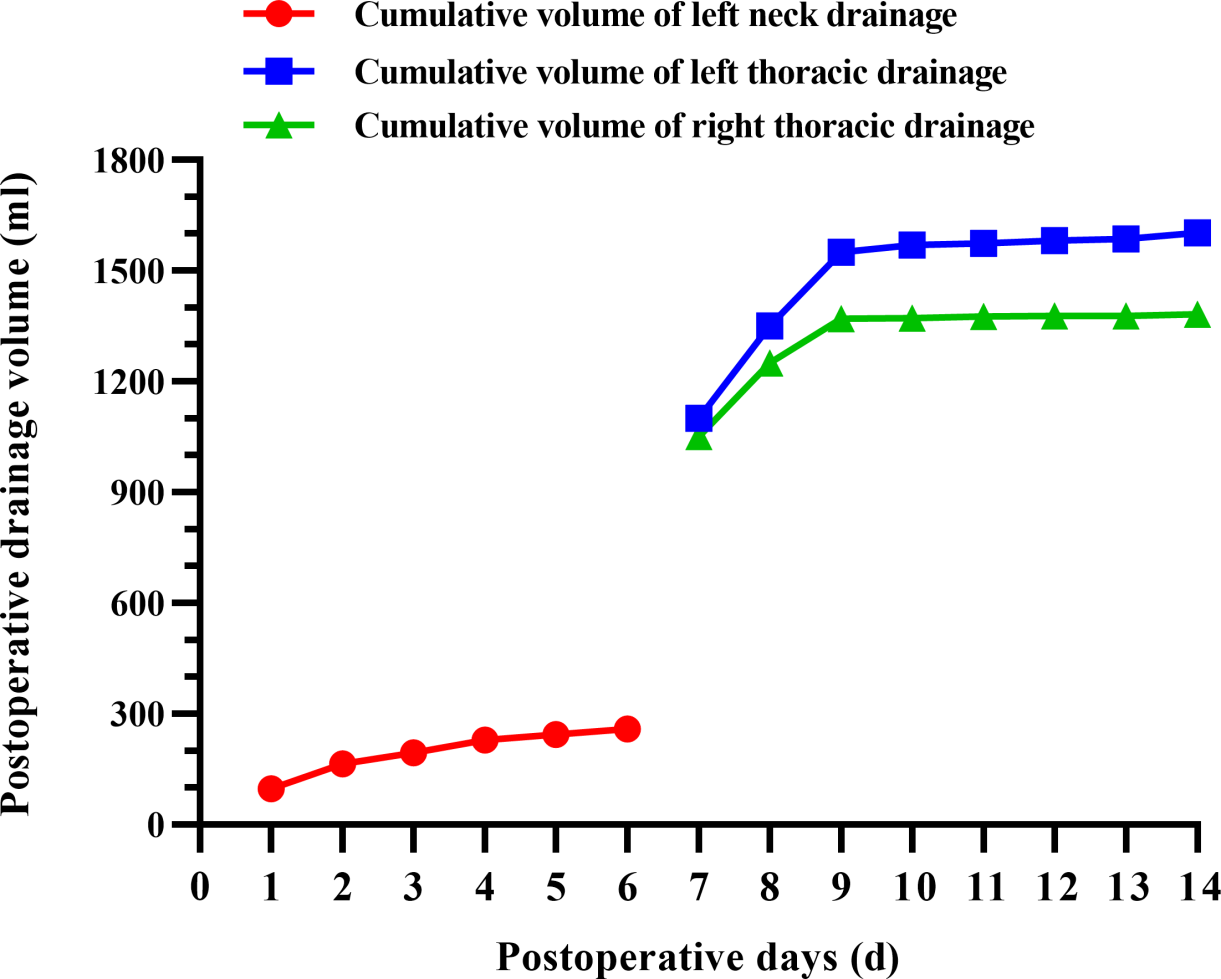
Graph of postoperative cervicothoracic drainage volume against time.

**Figure 4:**
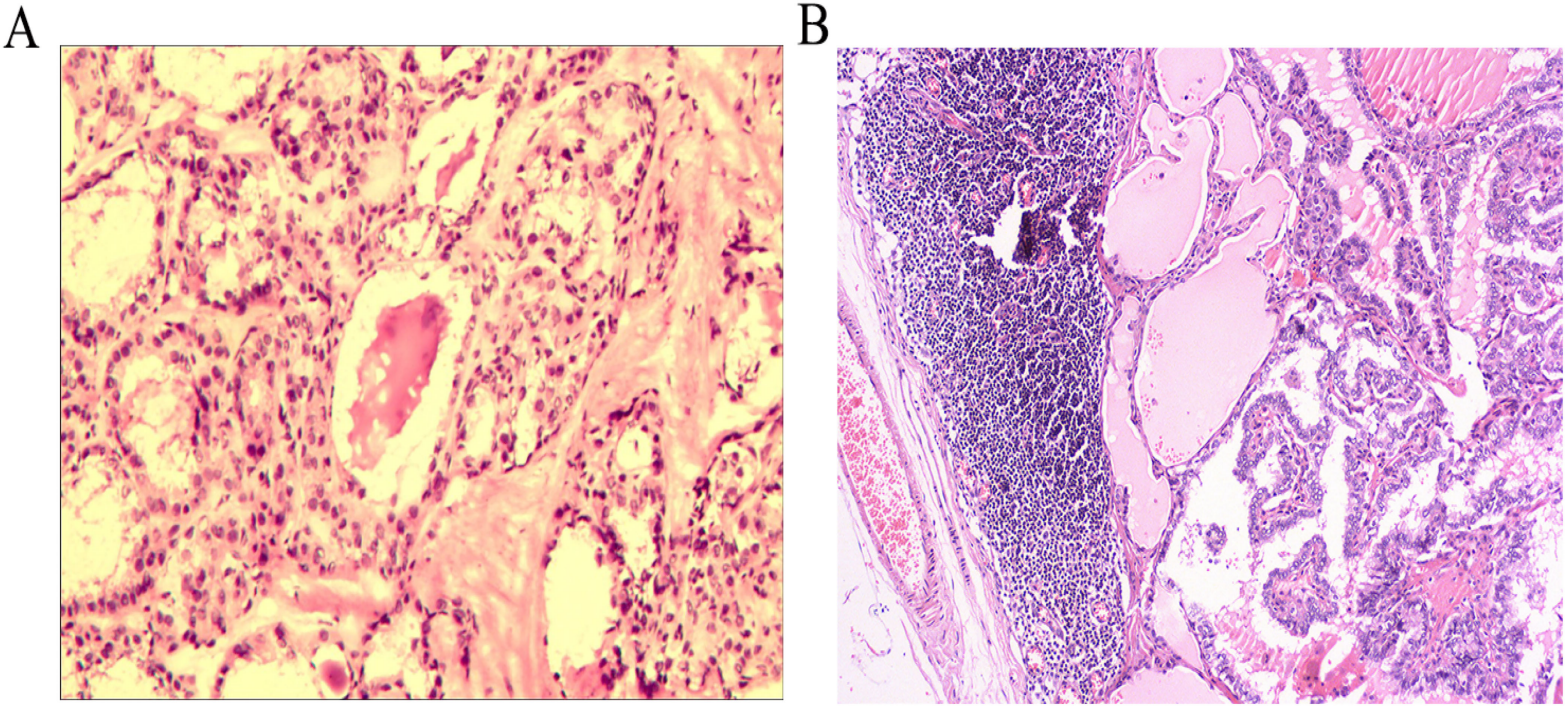
Postoperative pathological examination images of the thyroid **(A)**, metastatic lymph nodes in the left neck **(B)**.

The patient did not exhibit any specific discomfort immediately following the surgery and there was no chylous fluid outflow from the neck drainage tube. Subsequently, the neck drainage tube was removed on the fourth day. However, on the 7th day post-operative, the patient experienced chest tightness, shortness of breath, dyspnea, and decreased lung auscultation breath sounds. The chest X-ray examination revealed the presence of bilateral pleural effusion (**Figure 2A**). Consult the department of thoracic surgery, implanted two 16-French thoracic closed drainage tubes under ultrasound guidance(the thoracic closed drainage tubes were positioned in the 7th left intercostal space and the 8th right intercostal space). Approximately 2150mL of milky white fluid was discharged(1100 mL of left thoracic drainage, 1050 mL of right thoracic drainage), and pleural fluid analysis showed a triglyceride level of 4.5 mmol/L (normal range: 0.28–1.80mmol/L), yielded positive results. The patient is diagnosed with bilateral chylothorax. The patient was placed on conservative treatment (including fasting and parenteral nutrition) along with daily monitoring of electrolytes. In addition, electrolyte disorder are corrected by intravenous and oral electrolytes based on the corresponding laboratory results. On the 8th day following operative, the patient experienced a significant reduction in discomfort, including chest tightness, shortness of breath, and dyspnea. Additionally, there was a sudden decrease in drainage output, with only 450mL over a 24-hour period(250 mL of left thoracic drainage, 200 mL of right thoracic drainage). On postoperative day 9, 200 mL of left thoracic drainage, 120 mL of right thoracic drainage were reported. On postoperative day 10, the left thoracic drainage volume was reduced to 20 mL, and the right thoracic drainage volume to 2 mL. The bilateral pleural drainage volume was 4 mL on the 11th postoperative day. On postoperative day 12, 7 mL of left thoracic drainage, 2 mL of right thoracic drainage were reported. On postoperative day 13, 5 mL of left thoracic drainage and 2 mL of right thoracic drainage were obtained. On the 14th day post-operative, the patient exhibited minimal drainage from the chest tube(10 mL of left thoracic drainage, 4 mL of right thoracic drainage), and experienced complete relief of chest tightness and dyspnea. Subsequent reexamination revealed no accumulation of fluid in the chest on X-ray, leading to the removal of the closed thoracic drainage tube (**Figure 2B**). The postoperative cervicothoracic drainage volume is illustrated in **Figure 3**. The patient was placed on a low-fat diet and kept under observation for a period of 2 days. Following the dietary regimen(comprising fish, chicken, vegetables and rice), the patient exhibited no signs of discomfort post-meal and was subsequently discharged. The postoperative pathological examination revealed left papillary thyroid carcinoma(pT2N1bM0 stage I), multifocal distribution, measuring 3.5cm×2.5cm in maximum diameter. There was evidence of metastasis to the left central region (1/1) and left cervical lymph nodes (12/18) (**Figure 4**).

Literature review revealed that 50 cases of chylothorax occurred following neck lymph node dissection for thyroid cancer (6–28). There were 8 unilateral and 31 bilateral chylothorax. The primary clinical characteristics are outlined in **Table 1**.

**Table 1:** Characteristics of reviewed reports of chylothorax occurred following neck lymph node dissection for thyroid cancer.

Author	Number of cases	Age	Sex	Postoperative symptoms	Chylothorax appearance time(Postoperative day)	Examination	Chylothorax position	Therapy method	Curative effect
**Har-El (** 6)	1	34	Female	thoracodynia、dyspnea	3	Chest X-ray	Bilateral	Conservative treatment	Recovery
**Jiaxing Huang (** 7)	1	53	Male	chest tightness、shortness of breath	2	Chest X-ray	Right side	Conservative treatment	Recovery
**Jabbar AS (** 8)	1	35	Female	thoracodynia、dyspnea	4	Chest X-ray+chylothorax test	Bilateral	Conservative treatment	Recovery
**Kiyoaki Tsukahara (** 9)	1	72	Female	dyspnea	2	Chest X-ray	Bilateral	Conservative treatment	Recovery
**Ja Seong Bae (** 10)	2	46	Female	dyspnea	7	Chest X-ray+chest CT+chylothorax test	Bilateral	Conservative treatment	Recovery
		47		Chest discomfort、dyspnea	3		Bilateral	Conservative treatment	Recovery
**Himanshu Khurana (** 11)	1	17	Female	Chest discomfort、dyspnea	2	Chest X-ray	Bilateral	Conservative treatment	Recovery
**Han C (** 12)	1	42	Female	thoracodynia、dyspnea	8	Chest X-ray	Bilateral	Conservative treatment	Recovery
**Tallón-Aguilar (** 13)	1	38	Female	dyspnea	3	Chest X-ray	Bilateral	Conservative treatment	Recovery
**Zhonghua Zhou (** 14)	5	Unknown	Unknown	chest tightness, shortness of breath	2-3	Chest X-ray	Left side: 2; Bilateral: 3	Conservative treatment: 2; Conservative treatment+Surgical treatment: 3	Recovery
**Tian Wei (** 15)	3	40	Female	Chest discomfort、dyspnea	4	Chest X-ray+chylothorax test	Bilateral	Conservative treatment	Recovery
		38		Chest discomfort、dyspnea	4		Left side	Conservative treatment	Recovery
		31		Chest discomfort、dyspnea	3		Left side	Conservative treatment	Recovery
**Zhiyu Li (** 16)	5	48	Female	chest tightness	2	Chest X-ray	Bilateral	Conservative treatment	Recovery
		65		chest tightness、 dyspnea	8		Bilateral	Conservative treatment	Recovery
		40		Chest discomfort	2		Bilateral	Conservative treatment+Surgical treatment	Recovery
		38		Chest discomfort、dyspnea	4		Left side	Conservative treatment	Recovery
		31		Chest discomfort、dyspnea	3		Bilateral	Conservative treatment	Recovery
**Baoqing Li (** 17)	1	57	Male	dyspnea	1	Chest X-ray+chylothorax test	Left side	Conservative treatment	Recovery
**Detao Yin (** 18)	1	27	Female	chest tightness	4	Chest X-ray+chylothorax test	Bilateral	Conservative treatment	Recovery
**Tina Runge (** 19)	1	40	Female	Chest discomfort、dyspnea	2	Chest CT+chylothorax test	Bilateral	Conservative treatment	Recovery
**Xianjiang Wu (** 20)	11	21-67	Male:4; Female:7	chest tightness, shortness of breath	2-4	Chest X-ray	Unilateral or bilateral	Conservative treatment: 10; Conservative treatment+Surgical treatment: 1	Recovery
**Noriaki Hayashibara (** 21)	1	48	Female	dyspnea	4	Chest X-ray	Bilateral	Conservative treatment	Recovery
**Verena Merki (** 22)	1	54	Male	dyspnea	2	Chest CT+chylothorax test	Bilateral	Conservative treatment	Recovery
**Tiecheng Feng (** 23)	2	24	Female	shortness of breath, dyspnea	4	Chest X-ray	Bilateral	Conservative treatment	Recovery
		28		shortness of breath, dyspnea	4		Bilateral	Conservative treatment	Recovery
**Jingjing Shi (** 24)	6	29-75	Female	chest tightness, shortness of breath	2-4	Chest CT+chylothorax test	Bilateral	Conservative treatment	Recovery
**Qingzhuang Liang (** 25)	1	55	Female	chest tightness,thoracodynia	4	Chest X-ray+chylothorax test	Right side	Conservative treatment	Recovery
**Yongkang Li (** 26)	1	69	Female	cough、expectoration	2	Chest CT+chylothorax test	Bilateral	Conservative treatment	Recovery
**Musallam Kashoob (** 27)	1	35	Female	chest tightness, shortness of breath	7	Chest X-ray+chylothorax test	Bilateral	Conservative treatment	Recovery
**Wencong Sun (** 28)	1	28	Female	Chest discomfort、dyspnea	3	Chest CT+chylothorax test	Bilateral	Conservative treatment	Recovery

## Discussion

The left side of the neck contains the thoracic duct, while the right side contains the right lymphatic duct. These lymphatic ducts collect lymph fluid from throughout the body and eventually drain into the left and right venous angle. The thoracic duct is the largest lymphatic duct in the body, collecting approximately 75% of the body’s lymphatic fluid (29). Therefore, chylothorax is closely associated with issues related to the thoracic duct. The mechanism of chylothorax following cervical lymph node dissection remains unclear, and two hypotheses have been proposed (18). One hypothesis suggests that damage to the thoracic duct or the right lymphatic duct occurred during the dissection of venous angle lymph nodes during the operation, leading to cervical chylothorax. As a result, chylous fluid flowed from the root of the neck into the mediastinum and entered the pleural cavity under fluid pressure, ultimately causing chylothorax. Another hypothesis suggests that the routine ligation of lymphatic vessels at the root of the neck, aimed at preventing post-operative lymphatic leakage, may inadvertently lead to the ligation of the thoracic duct or right lymphatic duct. This could result in an obstruction of lymphatic return and increased pressure, potentially causing lymphatic fluid to overflow the lymphatic wall and accumulate in the pleural cavity when pressure reaches a certain threshold. In this case, the anesthesiologist inflated the lung by overinflating the ventilator after the operation and found no lymphatic effusion in the operative area. Additionally, the postoperative cervical drainage tube did not drain chylous fluid. However, the patient developed bilateral chylothorax. Therefore, it is speculated that the bilateral chylothorax in this patient may be attributed to abnormal pressure changes in the thoracic duct.

The diagnosis of chylothorax should be based on a combination of the patient’s clinical manifestations, chest imaging, and thoracentesis. Typical clinical symptoms include chest tightness and progressive dyspnea. The puncture fluid is generally milky white, but its properties are related to the fat content consumed by the patient. If the drainage volume is high, even if the color is light red, chylothorax should be considered and a chyle test can be performed. Hydrothorax can be diagnosed when the triglyceride content in pleural fluid is greater than 1mg/L and the cholesterol/triglyceride ratio is less than 1 (30). Therefore, the patient was diagnosed with bilateral chylothorax.

Conservative management of chylothorax involves timely thoracic catheterization and drainage, adherence to a low-fat, fat-free diet or fasting, provision of parenteral nutrition support, prevention of water and electrolyte imbalances, and prevention of pulmonary infection (31). Closed thoracic drainage is advantageous for rapidly alleviating pressure, relieving dyspnea and other symptoms, and for observing the nature and flow of drainage fluid to inform further treatment. Fasting or low-fat diet, as well as parenteral nutrition, can help alleviate the burden on the gastrointestinal tract and reduce the formation of chyle. In this case, following the conservative treatment mentioned above, the color of thoracic chylous fluid gradually lightened and the drainage volume decreased progressively. As a result, the patient was successfully discharged on the 14th day after surgery without experiencing any serious consequences. Surgical interventions, including thoracic catheter ligation, thoracic catheter embolization, and pleurodesis, should be considered if conservative treatment is not effective, chylous leakage≥1L/d persists for more than 5 days, or if serious complications exist (32–35). However, the effect of surgical treatment remains uncertain, and it is advisable to continue conservative treatment in patients.

We conducted a systematic review of 50 similar cases that have been reported in the past (6–28). All patients experienced chest discomfort (such as chest pain, shortness of breath, dyspnea, etc) approximately one week following the surgical procedure. When these symptoms manifest, patients underwent chest imaging examination or a chylothorax test to confirm the presence of chylothorax. Out of this total, 45 patients underwent conservative treatment, while the remaining 5 received a combination of conservative and surgical treatments. All patients recovered fully and were subsequently discharged from the hospital. In our case, the patient experienced chest discomfort on the 7th day post-operative. Subsequently, a chest X-ray and chylothorax test were conducted to diagnose chylothorax. The patient received conservative treatment and was later discharged from the hospital after a full recovery.

## Conclusion

Bilateral chylothorax after neck lymph node dissection for thyroid cancer is a rare complication. During the operation, it is important to avoid causing damage to the thoracic duct and right lymphatic duct. If a patient experiences chest tightness, shortness of breath, dyspnea, and other symptoms following surgery, the possibility of chylothorax should be considered. It is recommended to promptly conduct a chest X-ray or chest CT, especially when accompanied by inadequate neck drainage. Once chylothorax is diagnosed, initial conservative treatment should be pursued, with surgical intervention being considered if deemed necessary. In clinical practice, it is imperative to enhance our understanding of these conditions and to ensure timely detection and intervention in order to prevent serious or potentially fatal complications.

## Data Availability

The raw data supporting the conclusions of this article will be made available by the authors, without undue reservation.
